# Exploring innovation landscapes: a national cross-sectional study of Swedish primary care from the viewpoint of primary care managers

**DOI:** 10.1186/s12913-026-14870-y

**Published:** 2026-06-25

**Authors:** Sarah Samuelson, Eva-Lisa Petersson, Cecilia Björkelund, Marcus Praetorius Björk, Dominique Hange, Malin Östman, Sven Persson Kylén, Irene Svenningsson

**Affiliations:** 1 General Practice/Family Medicine, School of Public Health and Community Medicine, Institute of Medicine, Sahlgrenska Academy, University of Gothenburg, Gothenburg, Sweden; 2 Research, Education, Development & Innovation (REDI), Region Västra Götaland, Primary Health Care, Gothenburg, Sweden

**Keywords:** Primary care, Innovation, Collaboration, Healthcare management, Healthcare policy, Digitalisation, Cross-sectional study, Mixed methods.

## Abstract

**Background:**

Innovation is increasingly necessary in primary care policy, and healthcare managers play a pivotal role in translating policy ambitions into practice and shaping organisational conditions that foster innovation. The aim of this study was to explore innovation activities and their contextual conditions in Swedish primary care, as perceived by primary care managers.

**Methods:**

A national cross-sectional mixed-methods survey was conducted across Swedish primary care centres. Quantitative data were analysed using descriptive statistics, while qualitative data were analysed using systematic text condensation.

**Results:**

In total, 392 of the 1,028 invited primary care managers responded to the questionnaire. Approximately 80% of primary care managers reported product, process or organisational innovations, reflecting relatively high innovation activity at primary care centres. Innovation ideas predominantly emerged from within the primary care centre, and managers viewed themselves as playing a central role in generating these ideas. Most innovation activities involved internal collaboration between staff groups (86%) and units (62%), with limited external collaboration (6%–12%). Leadership was central in engaging staff with innovation and cultivating an innovation-supportive culture. Managers provided time, space and organisational support for continuous competence development. However, organisational and resource constraints limited staff involvement in innovation, as primary care remains tightly bound to economic and production-related demands.

**Conclusion:**

This study offers an overview of the innovation landscape in Swedish primary care from the perspective of primary care managers and contributes empirical knowledge on how innovation is understood and managed across different primary care contexts in Sweden. While Swedish primary care demonstrates strong engagement in innovation, the involvement of and collaboration with external stakeholders remain limited, underscoring the need for system-level strategies and processes to facilitate cross-sector innovation partnerships. Further research is needed on how system-level frameworks for collaboration and innovation can be established across the Swedish healthcare system, including primary care.

**Supplementary Information:**

The online version contains supplementary material available at 10.1186/s12913-026-14870-y.

## Background

In recent years, innovation has gained prominence in healthcare policy and is increasingly promoted as a strategy to increase efficiency, improve patient-centredness and accelerate digital transformation [1–5]. This emphasis reflects broader systemic pressures, including demographic shifts, rising demand for services and constrained resources, which render innovation not merely desirable but essential [6]. Sweden is no exception, as successive reforms and policy frameworks have consistently positioned innovation at the core of primary care. National strategies emphasise the role of digitalisation and innovation, including new models of service delivery and organisational development, as means of achieving more accessible and sustainable healthcare [7]. Moreover, there is a call to update legislation to further reinforce healthcare’s innovation mandate, which would include revising the Health and Medical Services Act to explicitly recognise innovation as part of the healthcare mission, supported by clearer mandates, requirements and responsibilities [8].

Innovation can be broadly understood as the process of combining knowledge, ideas and resources in new ways to address needs more effectively and efficiently [9]. In a healthcare context, innovation may take the form of new ideas, products, services, work practices, processes, organisational models or system-level changes [5, 10–12]. Crucially, an innovation is not only the generation of something new but also involves implementation and diffusion, leading to the replacement or improvement of existing solutions [13]. In the healthcare sector, innovation is often incremental and embedded in everyday practice rather than being characterised by radical technological breakthroughs [10]. In primary care, such innovation is often closely aligned with quality improvement and service development, which emerge from daily clinical and organisational challenges [14], illustrating how primary care adapts to evolving clinical and organisational needs. Previous research has demonstrated a wide range of innovations aimed at transforming practices, increasing efficiency and improving health outcomes in primary care, including new models of care [15, 16], clinical decision-support systems [17, 18], remote patient monitoring [19] and telemedicine platforms [20].

Thus, in the context of evolving healthcare demands, innovation has become essential for improving quality and efficiency. Yet, introducing new solutions or ways of working often proves challenging [21–24], as innovation requires transformations of practice. Advancing innovation in primary care therefore calls for a cultural shift that embraces change and supports service redesign [5, 11, 25]. Healthcare managers are central to this process, as they can foster innovative practices through sustained organisational strategies that build professionals’ innovative capacities and create environments conducive to learning and experimentation [14, 25, 26]. Inclusive workplaces, where staff feel valued and empowered, strengthen commitment and encourage idea-sharing, which are critical for driving innovation [26]. In contrast, rigid hierarchies and low perceived involvement in innovation activities among healthcare staff remain key obstacles [27].

Given the strong policy ambitions and growing expectations for innovation in Swedish primary care, as well as the key role managers play in translating policy into practice [28], there is limited national-level knowledge about how innovation activities are organised and experienced by primary care managers. Moreover, little empirical knowledge is available regarding how primary care managers perceive innovation and the factors influencing how innovation is supported, prioritised and negotiated within routine Swedish primary care practice. This knowledge gap hinders the development of effective organisational strategies, policies and support initiatives for innovation in primary care. The aim of this study was therefore to explore innovation activities and their contextual conditions in Swedish primary care, as perceived by primary care managers.

## Methods

### Study design and settings

We conducted a cross-sectional study using a mixed-methods approach. The study was carried out as a national online survey targeting primary care managers across Swedish primary care centres (PCCs). A cross-sectional study captures data from a population at a single point in time; it is useful for estimating the prevalence of conditions, generating hypotheses and describing relationships between variables, but it cannot establish causality [29]. A 2-year retrospective timeframe (2022–2023) was chosen to capture innovation activities that typically require time for implementation and initial assessment, while acknowledging the potential for recall bias.

The study employed a mixed-methods approach with an embedded design, in which one dataset (qualitative data) was used to complement the primary data source (quantitative data) [30]. Qualitative and quantitative data were collected simultaneously through a single questionnaire containing both closed- and open-ended questions. Each dataset was analysed separately to maintain methodological rigor, and the findings were then integrated during interpretation [30]. Quantitative data identify overarching patterns and relationships, while qualitative data offer depth and contextual understanding. Merging the two strengthens interpretations and enables validation across datasets [30, 31]. ChatGPT-5.2 (OpenAI) was used as a writing aid to improve the linguistic quality of the manuscript.

### Questionnaire

A modified version of Statistics Sweden’s (SCB) Innovations in Healthcare questionnaire was used. The original questionnaire had previously been administered in a national survey of hospital and primary care settings in 2014 [32]. However, as we only had access to the version used in hospital settings, minor contextual adaptations were made to ensure relevance to primary care; for example, terms such as ‘hospital’ were replaced with ‘primary care’ or ‘primary care centre’. In addition, supplementary background questions were included, and the wording and instructions were partially revised to align with updates to the SCB’s *Public Sector Innovation Survey* [33], while preserving the original content and purpose of the questions.

To further explore the contextual conditions for innovation, an open-ended question on how managers promote employee engagement in innovation was added. However, to balance the length of the questionnaire and minimise respondent burden, the section on communicative innovations was omitted. All modifications were carried out in consultation with a senior researcher experienced in primary care innovation (IS) and were reviewed by the research team (ELP, DH, CB, SPK, MÖ).

The questionnaire comprised 30 items, primarily consisting of closed-ended multiple-choice questions (e.g. yes/no/do not know; to a large extent/to some extent/not at all/do not know), with a few open-ended questions. Some closed-ended items allowed respondents to provide a written response if none of the options were applicable. In addition to background questions (*n* = 8), the questionnaire addressed the following areas: types of innovation (product, process and organisational) (*n* = 4); effects of innovation (*n* = 1); support for innovation (*n* = 2); drivers and strategies for innovation (*n* = 6); innovation culture and organisation (*n* = 3); the PCC’s most significant innovation (*n* = 5); and conditions for staff engagement in innovation (*n* = 1). On average, completion took 17 min. This study focuses on innovation activities and their contextual conditions; therefore, data on innovation effects and the PCC’s most significant innovation are not reported. An English translation of the full questionnaire is provided in Additional file 1.

### Recruitment, study population and data collection

Recruitment and data collection took place during the first half of 2024. First, email addresses for all primary care managers in Sweden were compiled (*n* = 1345). The inclusion criterion was holding a managerial position at a public or private PCC. Facilities such as branches of a PCC under the same manager, urgent or out-of-hours clinics, vaccination clinics and centres providing only certificates, vaccinations, general health checks or occupational health services were excluded (*n* = 191). These exclusions were applied to reduce the likelihood of the same manager receiving multiple questionnaires and to ensure that the study focused on conventional PCC services. Initial contact was made with all regional authorities (*n* = 21) and major private providers (*n* = 8) to request manager contact information. Addresses not provided were obtained from the national healthcare portal 1177 or the PCC’s websites. When necessary, supplementary searches were conducted using public sources such as LinkedIn and previous job advertisements. The managers received the study introduction letter and questionnaire digitally via the survey and analysis platform esMaker. Reminder letters were sent to non-respondents after 1, 2 and 4 weeks, for a total of three reminders.

**Fig. 1:**
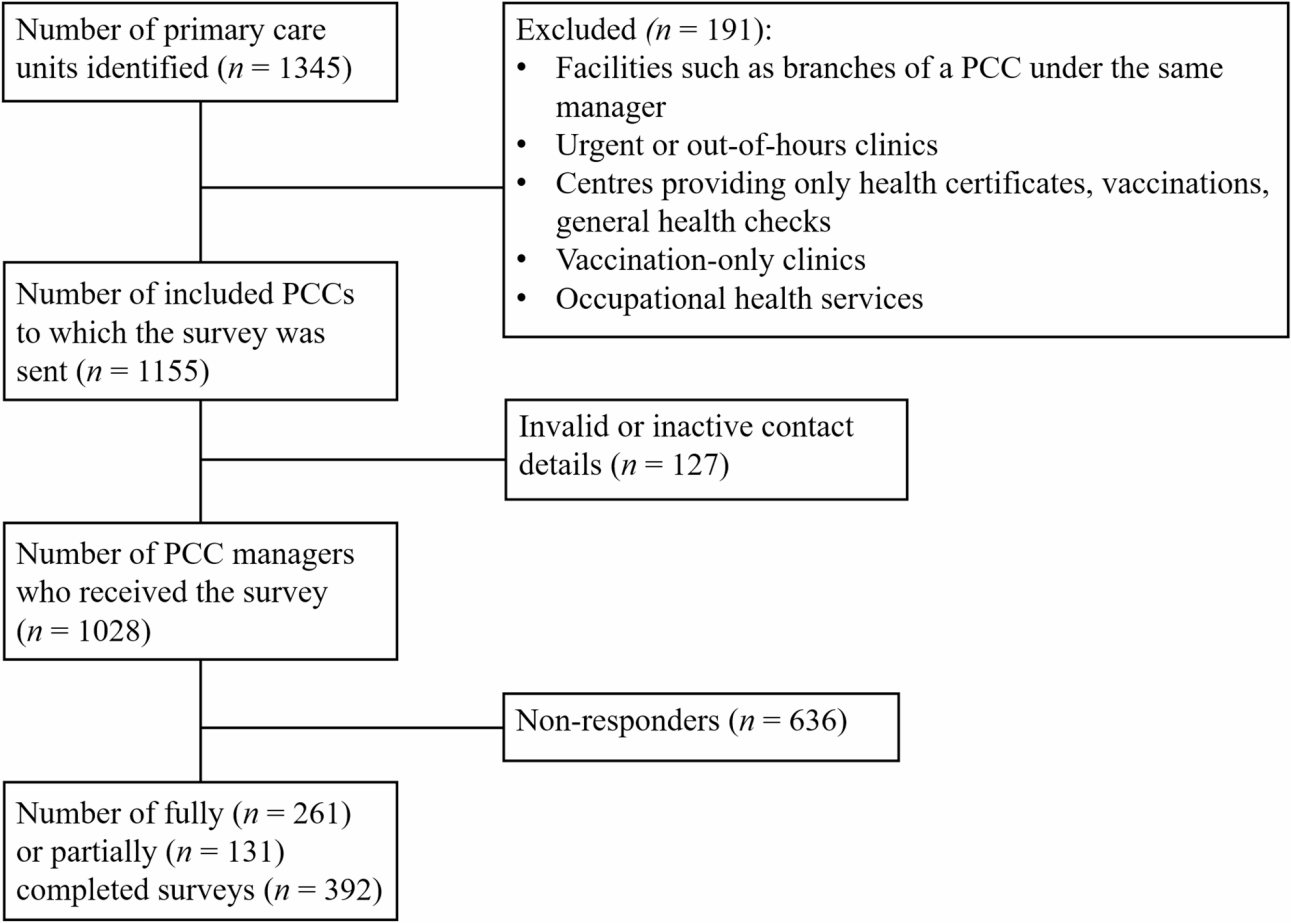
Flow of the survey distribution and responses among primary care managers

The survey was distributed to a total of 1155 managers. Of these, 127 invitations could not be delivered due to invalid or inactive contact details (e.g. wrong recipient, terminated position or prolonged absence). Consequently, 1028 managers successfully received the questionnaire. The proportion of undeliverable invitations corresponded to an external non-response rate of 11% (127/1155). In total, 392 managers responded to the survey, corresponding to an overall response rate of 38% (392/1028). Of these, 261 managers completed the questionnaire in full, while 131 provided partial responses, resulting in a completion rate of 25% (261/1028) for fully completed questionnaires (Fig. 1). Response rates were highest for the initial questions and gradually declined. The number of responses for each question is presented in the corresponding tables in the results section.

### Non-response analysis

Respondents who chose not to participate were asked to provide feedback on their reasons for non-participation or partial participation. In total, 59 individuals responded, some of whom provided multiple reasons. The main reason for non-response was lack of time and competing priorities (*n* = 51). Other reasons included being a new manager or working in a newly established PCC (*n = *12); difficulty answering the questions (*n* = 8); perceived irrelevance due to limited innovation activity (*n* = 3); and regional focus, with limited engagement in surveys initiated outside the region (*n* = 6). A table summarising managers’ reported reasons for non-response or partial response is provided in Additional file 2.

## Data analysis

### Quantitative analysis

#### Statistical analysis

Descriptive statistics were applied, and categorical variables are presented herein as frequencies and percentages. The managers’ years in their current position were categorised into four groups, ranging from < 2 years to > 10 years, and their professional backgrounds were classified into four domains. The sizes of the PCCs were initially divided into five categories according to the number of registered patients, ranging from < 5000 to > 20 000. For the analyses, however, the PCCs were dichotomised into two size categories: <10 000 or ≥ 10 000 registered patients. The locations of the PCCs were categorised as urban, rural or remote. Urban areas were defined as locations with more than 3000 inhabitants, including areas within a 5-minute drive to an urban centre. Rural areas were defined as locations within a 5- to 45-minute drive to an urban centre with more than 3000 inhabitants. Remote areas were defined as locations more than 45 minutes’ drive from the nearest urban centre with more than 3000 inhabitants, including islands without a fixed land connection [34]. For analytical purposes, PCCs were classified as either urban or non-urban, with the latter comprising both rural and remote areas.

Chi-square tests were used to examine associations between innovation type and PCC characteristics, including location, ownership type, PCC size, and the managers’ and employees’ academic level. Fisher’s exact test was applied when the assumptions for the chi-square test were not met. An initial significance level of α = 0.05 was applied and adjusted to α = 0.000357 using the Bonferroni correction to account for multiple comparisons. Written responses provided by managers when none of the predefined options applied were not quantified; instead, they were included in the analysis as examples of alternative answers. As the questionnaire consists of stand-alone items that were analysed independently, both complete and partially completed questionnaires were included in the analysis. However, questionnaires in which only background information was provided were excluded, and those managers were classified as non-responders. Statistical analyses were performed using IBM SPSS Statistics (version 28.0.1.1).

### Qualitative analysis

A qualitative analysis was used to explore how primary care managers create conditions for staff participation in workplace innovation and development. As the data consisted of written free-text responses with limited depth and contextual richness compared with interview data, a manifest analytical approach was considered most appropriate. Malterud’s systematic text condensation (STC) [35] was therefore chosen as the method of analysis. The analysis followed four iterative steps. First, all responses were repeatedly read to gain an overall impression and identify preliminary themes. Second, relevant meaning units were extracted and organised into thematic code groups. During this step, codes were further refined into subcodes to capture variations and nuances within the material. Third, the content of each code and subcode was condensed as required (since many meaning units were already concise), producing condensates that reflected the essence of the participants’ accounts. Finally, these condensates were recontextualised and integrated into an analytical text, resulting in descriptions supported by illustrative quotations. The data analysis was conducted by the first author (SS). To enhance the trustworthiness of the analysis, the development of codes and subcodes was reviewed and discussed with two senior researchers with extensive primary care experience (IS, ELP) until consensus was reached [36]. Additional file 3 presents an illustrative example of the analytical steps.

## Results

The study’s results are presented as follows. First, the quantitative findings are reported, beginning with a description of the participant and PCC characteristics. This is followed by innovation types, support for innovation, drivers and strategies, ending with innovation culture and organisation. The qualitative findings are then presented under the codes of *leadership and culture*, *collaboration and learning*, and *structure and processes*.

### Quantitative results

#### Participant and PCC characteristics

The majority of respondents were primary care managers (93%), most of whom had a healthcare professional background (88%) and had held their current position for 2–5 years (43%). Just over half of the PCCs were publicly owned, and most were located in urban areas. PCC size varied, with the majority serving between 5 000 and 14 999 registered patients. Detailed characteristics are presented in Table 1.

**Table 1:** Participants and PCC characteristics

Participants and PCC characteristics	*n* (%)
Managerial position	
Primary care manager Deputy primary care manager Other (e.g. first-line manager, department manager)	363 (92.8) 5 (1.3) 23 (5.9)
Years in current managerial position	
<2 2–5 6–10 >10	105 (26.9) 168 (43.0) 79 (20.2) 39 (10.0)
Professional background	
Healthcare profession Psychosocial and behavioural profession Administrative and managerial profession Technical and development-oriented profession	342 (87.7) 25 (6.4) 18 (4.6) 5 (1.3)
Manager with a PhD	
Yes No	22 (5.6) 370 (94.4)
Employee with a PhD	
Yes, throughout the entire period 2022–2023 Yes, during parts of the period 2022–2023 No	115 (29.3) 33 (8.4) 244 (62.2)
Location of PCC	
Urban area Rural area Remote area	298 (76.0) 58 (14.8) 36 (9.2)
Ownership type	
Publicly owned Privately owned Other	214 (54.6) 177 (45.2) 1 (0.3)
Number of registered patients	
<5000 5000–9999 10 000–14 999 15 000–20 000 >20 000	57 (14.5) 182 (46.4) 122 (31.1) 24 (6.1) 7 (1.8)

#### Innovation type

Innovations at the PCCs in 2022–2023 were categorised as product, process or organisational. To qualify, innovations did not have to be new to other PCCs, hospitals, companies or organisations, but they had to differ significantly from previous practices within the PCC and be implemented for users. Overall, approximately four out of five managers reported the introduction of product, process and organisational innovations during 2022–2023 (Table 2). No significant differences were observed with respect to ownership, geographical location, size or the presence of an employee or manager with a PhD.

**Table 2:** Overview of product, process and organisational innovation

		Observed	Not observed	Public/private	Urban/non-urban	Size < 10 000/ ≥10 000	Employee with/without a PhD	Manager with/without PhD
Type of innovation	*n*	*n* (%)	*n* (%)	*p* value	*p* value	*p* value	*p* value	*p* value
Product innovation	392	331 (84.4)	61 (15.6)	0.074	0.066	0.097	0.571	<0.001**
Process innovation	392	324 (82.7)	68 (17.3)	0.258	0.400	0.130	0.853	0.779
Organisational innovation	392	312 (79.6)	80 (20.4)	0.843	0.408	0.278	0.179	0.411

*Statistically significant after Bonferroni correction for multiple comparisons

**Although p < 0.001, the result is not significant after Bonferroni correction

##### Product innovations

Product innovations include new or improved goods and services offered by the PCC (Table 3). The three most common product innovations were services used by patients or other citizens (67%), medical equipment and instruments (53%) and medical treatments (36%). The results indicate that public PCCs were more likely to introduce product innovations that were new both to the PCC and to primary care in general (*p* < 0.001).

##### Process innovations

Process innovations are new or improved ways of working within one or more functions of the PCC (Table 3). The three most common process innovations were methods to reduce waiting times (75%), guidelines for coordinating care around the individual patient (69%) and treatment programmes or therapeutic strategies (59%).

##### Organisational innovations

Organisational innovations involve the introduction of new or improved organisational methods within the PCC, arising from strategic decisions made by its management (Table 3). The three most common organisational innovations were other methods for organising work (78%), collaboration between municipalities and regions (61%) and measures to reduce the administrative burden on healthcare staff (59%).

**Table 3:** Overview of product, process and organisational innovations introduced in 2022–20232

Types of innovation introduced in 2022–2023	*n*	Yes	No	Do not know	Public/ private	Urban/ non-urban	PCC size < 10.000/ ≥ 10.000	Employee with/without PhD	Manager with/without PhD
		*n* (%)	*n* (%)	*n* (%)	*p* value	*p* value	*p* value	*p* value	*p* value
*Product innovation*									
Medical treatments	391	141 (36.1)	203 (51.9)	47 (12.0)	0.651	0.224	0.004	0.135	0.776
Medical equipment and instruments	392	206 (52.6)	162 (41.3)	24 (6.1)	0.235	0.033	0.476	0.211	0.419
Non-medical equipment and instruments	390	122 (31.3)	214 (54.9)	54 (13.8)	0.088	0.419	0.503	0.625	0.556
Services used by patients or other citizens	390	263 (67.4)	97 (24.9)	30 (7.7)	0.032	0.277	0.735	0.989	0.517
Other services or goods	390	95 (24.4)	209 (53.6)	86 (22.1)	0.315	0.277	0.761	0.892	0.957
Development of product innovations not yet introduced	390	99 (25.4)	254 (65.1)	37 (9.5)	0.277	0.365	0.476	0.664	0.542
Product innovations new only to the PCC	390	175 (44.9)	183 (46.9)	32 (8.2)	0.014	0.062	0.856	0.691	0.795
Product innovations new to both the PCC and primary care at large	390	153 (39.2)	190 (48.7)	47 (12.1)	<0.001* §public	0.007	0.577	0.611	0.707
*Process innovation*									
Treatment programs or therapeutic strategies	347	204 (58.8)	122 (35.2)	21 (6.1)	0.982	0.914	0.163	0.605	0.069
Diagnostic methods	347	115 (33.1)	189 (54.5)	43 (12.4)	0.353	0.857	0.378	0.146	0.060
Guidelines for coordinating care around the individual patient	348	240 (69.0)	94 (27.0)	14 (4.0)	0.689	1.000	0.330	0.413	0.336
Methods for involving patients/relatives in decision-making	348	122 (35.1)	200 (57.5)	26 (7.5)	0.752	0.424	0.932	0.932	0.877
Methods for engaging patients in their own care	348	191 (54.9)	135 (38.8)	22 (6.3)	0.439	0.462	0.600	0.187	1.000
Methods for reducing waiting times	348	261 (75.0)	74 (21.3)	13 (3.7)	0.077	0.241	0.193	0.569	0.226
Support services (maintenance, procurement, logistics, etc.)	347	106 (30.5)	202 (58.2)	39 (11.2)	<0.001**	0.732	0.612	0.532	0.036
Other methods for the production of services and goods	347	65 (18.7)	198 (57.1)	84 (24.2)	0.006	0.674	0.435	0.996	0.821
Development of process innovations not yet introduced	347	119 (34.3)	192 (55.3)	36 (10.4)	0.928	0.494	0.706	0.545	­0.676
*Organisational innovation*									
Organisation of work responsibilities or decision-making	331	190 (57.4)	126 (38.1)	15 (4.5)	0.002	0.151	0.632	0.103	0.046
Organisational management systems for increased efficiency and improved outcomes (e.g. Lean)	331	124 (37.5)	197 (59.5)	10 (3.0)	0.119	0.706	0.184	0.806	0.125
Systems for gathering and processing knowledge and information (e.g. quality registers)	331	139 (42.0)	181 (54.7)	11 (3.3)	0.010	0.887	0.746	0.555	0.127
Education or training systems for staff or management	331	124 (37.5)	187 (56.5)	20 (6.0)	0.065	0.880	0.922	0.522	0.057
Measures to reduce the administrative burden on healthcare staff	331	195 (58.9)	124 (37.5)	12 (3.6)	0.026	<0.001**	0.052	0.350	0.196
Collaboration between municipalities and regions	331	202 (61.0)	112 (33.8)	17 (5.1)	0.962	0.251	0.420	0.858	0.376
Other methods for organising work (e.g. new ways of working)	331	257 (77.6)	58 (17.5)	16 (4.8)	0.355	1.000	0.468	0.915	0.276
Development of organisational innovations not yet introduced	331	124 (37.5)	185 (55.9)	22 (6.6)	0.854	0.798	0.931	0.118	0.338

*Statistically significant after Bonferroni correction for multiple comparisons

** Although *p* < 0.001, the result is not significant after Bonferroni correction

§ In the *p*-value columns, the group with the highest proportion (‘Yes’) is indicated

#### Support for innovation

Approximately one third of the PCCs received external support from municipal or regional authorities. Support from other actors – such as government agencies, the EU, other international sources, and private foundations or interest organisations – was generally limited (2%–10%). Additional file 4 provides a table presenting the support for innovation.

#### Drivers of and strategies for innovation

##### Drivers of innovation

According to the managers, innovations were most commonly initiated in response to staff requests (81%), the introduction of new regional policies or priorities (45%) and the need to implement new information technology (IT) systems (45%). In the open-ended comments, managers noted that internal challenges (e.g. high workloads, long waiting lists and limited accessibility) and demographic trends – especially the growing population of older people – require innovation and new working methods. Interest in innovation and quality improvement among managers and staff was also commonly reported as a driving force in the open-ended responses. However, requests from healthcare staff were the main reason for initiating innovations (Table 4).

##### Originators of innovation ideas

Managers reported that ideas for innovations most often originated within their own PCC, from management (82%), nurses (66%) and physicians (65%). PCC management, including the primary care manager, was identified as the primary actor in idea generation (42%). However, in the open-end comments, several managers emphasised that innovation ideas often stem from the entire team, rather than from a single professional group. Ideas originating from outside the primary care organisation were limited, with few reported to come from universities or public research organisations (6%) or private companies (8%). Patient and relative contributions accounted for 19% (Table 4).

##### Collaboration for innovation

Innovation-related collaboration was mainly internal, occurring primarily between staff groups at the PCC (86%), between units within the PCC (62%) and, to a lesser extent, with other PCCs and healthcare organisations (48%). The most important innovation collaboration was reported between staff groups at the PCC (61%). Collaboration with individual patients or external partners, such as patient organisations, industry, academia and other non-profit or non-governmental organisations, was limited (6%–12%) (Table 4).

**Table 4:** Drivers of and strategies for innovation

Drivers of and strategies for innovation	*n*	Yes *n* (%)	No *n* (%)	Do not know *n* (%)	Main reason (%)
*Which of the following reasons contributed to innovations being initiated at the PCC during 2022–2023?*					
Requests from patients/relatives or patient organisations	273	76 (27.8)	171 (62.6)	26 (9.5)	13.1
Requests from healthcare staff	273	222 (81.3)	37 (13.6)	14 (5.1)	36.7
Introduction of new laws or regulations	272	94 (34.6)	156 (57.4)	22 (8.1)	6.7
Introduction of new regional policies or priorities	272	123 (45.2)	123 (45.2)	26 (9.6)	6.7
Problem or crisis requiring immediate action	272	100 (36.8)	155 (57.0)	17 (6.3)	7.9
Requirement to implement new IT systems	272	122 (44.9)	136 (50.0)	14 (5.1)	7.1
Innovations at other PCCs	272	98 (36.0)	151 (55.5)	23 (8.5)	1.5
Internal reorganisation	272	119 (43.8)	139 (51.1)	14 (5.1)	5.2
Reduction of the PCC’s budget	272	81 (29.8)	171 (62.9)	20 (7.4)	6.0
Increase of the PCC’s budget	272	11 (4.0)	242 (89.0)	19 (7.0)	0.4
Other	272	36 (13.2)	128 (47.1)	108 (39.7)	8.6
*Which of the following actors came up with the ideas for the innovations at the PCC?*					*Most important actor (%)*
Primary care organisation management	272	123 (45.2)	135 (49.6)	14 (5.1)	19.1
PCC management	272	222 (81.6)	39 (14.3)	11 (4.0)	41.9
Physicians	272	176 (64.7)	81 (29.8)	15 (5.5)	7.5
Nurses	272	180 (66.2)	80 (29.4)	12 (4.4)	12.4
Assistant nurses	272	114 (41.9)	142 (52.2)	16 (5.9)	0.4
Rehabilitation staff	272	79 (29.0)	174 (64.0)	19 (7.0)	0.7
Other healthcare staff	272	65 (23.9)	184 (67.6)	23 (8.5)	1.1
Administrative staff	272	105 (38.6)	149 (54.8)	18 (6.6)	1.1
Service staff (e.g. cleaning, maintenance)	272	5 (1.8)	250 (91.9)	17 (6.3)	0.4
Patients or relatives	272	52 (19.1)	202 (74.3)	18 (6.6)	3.7
Other PCCs	272	74 (27.2)	177 (65.1)	21 (7.7)	1.5
Private companies	272	23 (8.5)	230 (84.6)	19 (7.0)	0.4
Universities or public research organisations	272	16 (5.9)	234 (86.0)	22 (8.1)	0.4
Regional politician	272	61 (22.4)	190 (69.9)	21 (7.7)	6.0
Other	272	16 (5.9)	182 (66.9)	74 (27.2)	3.4
*Did your PCC collaborate on innovations with any of the following actors during 2022–2023?*					*Most important actor (%)*
Units within your PCC	271	169 (62.4)	86 (31.7)	16 (5.9)	17
Staff groups at your PCC	271	232 (85.6)	28 (10.3)	11 (4.1)	61.6
Other PCCs and healthcare organisations	271	129 (47.6)	131 (48.3)	11 (4.1)	10.3
Patient organisations	271	24 (8.9)	234 (86.3)	13 (4.8)	0
Other non-profit or non-governmental organisations	271	15 (5.5)	238 (87.8)	18 (6.6)	1.1
Universities or public research organisations	271	32 (11.8)	225 (83.0)	14 (5.2)	2.6
Industry, consultants or business organisations	271	20 (7.4)	234 (86.3)	17 (6.3)	1.1
Individual patients	271	26 (9.6)	231 (85.2)	14 (5.2)	0.4
Other	271	20 (7.4)	198 (73.1)	53 (19.6)	5.9

#### Innovation culture and organisation

Just over half of the managers had specific objectives for innovation activities during 2022–2023 (51%) and had assigned individuals to drive innovations from idea to implementation (52%). However, fewer managers reported having a system to evaluate and develop the innovative ideas proposed by employees (38%) or a system to evaluate and introduce new medicines or treatments (23%). A majority of managers (61%) reported a lack of sufficient resources (e.g. time, funding and expertise) to develop an innovation. Just under one-third of PCCs (32%) involved patients and relatives in identifying problems and potential solutions, and 24% involved them in testing and implementing innovations. Additional file 5 provides a table presenting innovation culture, organisation and patient involvement in innovation.

Primary care managers regarded a change-oriented staff as by far the most important condition facilitating innovation activities (66%). Change-oriented primary care management at the administrative level and effective collaboration with other PCCs were also identified as facilitating factors, albeit to a considerably lesser extent (24% and 21%, respectively). The conditions most frequently reported as not having contributed at all to facilitating innovation activities included effective coordination with local and regional authorities (57%), effective collaboration with hospital care (57%) and change-oriented primary care management at the political level (41%). A table presenting conditions for innovation work is provided in Additional file 6.

### Qualitative results

A total of 216 managers responded to the open-ended question, ‘As a primary care centre manager, how do you create conditions for your staff to be involved in innovation and development at the workplace?’. Most responses were short and concise, while some offered more detailed descriptions. Three managers stated that they did not know. The analysis resulted in three codes: *leadership and culture*, *collaboration and learning*, and *structure and processes.*

#### Leadership and culture

Managers aimed to foster staff engagement in innovation and to cultivate an innovation-supportive culture through their leadership. Several managers stated that they valued innovation and development, and many sought to encourage employee involvement by inviting ideas and showing interest in employees’ suggestions. Another manager pointed out that the manager’s own academic and practical experience in improvement and development work formed a solid foundation for driving innovation. Moreover, the managers emphasised transparent and trust-based leadership, delegated responsibility and employee influence. Keeping staff informed of quality results and setting clear goals with regular follow-ups were also considered important.

…[I] place quite a lot of responsibility on individuals and teams to develop their respective areas. They are responsible for organising themselves and allocating their own time. (Public non-urban PCC with > 10 000 registered patients).

Several managers stressed the importance of staff recognising the need for and value of innovation and improvement, as the managers sought to motivate the staff through personal engagement, feedback on incentives and reflection on the impact of changes.

Openly presenting results from various measurements […] (patient surveys, number of registered patients, waiting times…) and discussing them – results that were not particularly flattering before the improvement work – to get staff to recognise for themselves the need for and value of improvement efforts. (Private urban PCC with < 10 000 registered patients).

Managers also pointed out the importance of being able to reconsider an innovation that does not appear valuable or falls short of expectations. One manager described the implementation of a digital-physical workflow. Although the initial rollout went well, increased accessibility led to higher workloads. Over time, this became their most significant work environment challenge, necessitating a step back to prevent burnout and staff turnover. The manager concluded,

…one should experiment with new approaches, but without becoming overly dogmatic or pursuing something at any cost merely to appear modern or follow trends. It is equally important to be able to step back when necessary. (Private urban PCC with > 10 000 registered patients)

Moreover, it was considered crucial to maintain a supportive and open environment that fosters experimentation and tolerates failure. Managers emphasised the importance of challenging established traditions and organisational culture, promoting continuous dialogue about methods and practices, encouraging teamwork and ensuring that all staff voices are valued.

#### Collaboration and learning

Some managers identified collaboration and learning as one way to promote staff involvement in innovation. One manager emphasised that innovation required a shared understanding and joint decision-making across professional groups to ensure new practices supported both patient care and sustainable working conditions for all staff at the PCC. Moreover, several managers described creating space, time and organisational support for continuous competence development. This included education and training activities, reflective forums, cross-professional dialogue and exposure to new ideas both within and beyond the organisation. Some also highlighted the importance of opening the organisation to wider networks – that is, other practices, municipal actors and research environments – to introduce fresh perspectives and specialised expertise.

Supporting staff with an interest in research and innovation was also noted, as staff engagement could stimulate wider participation and strengthen the PCC’s capacity for ongoing learning and development.

We have several employees who are driven by research and innovation. By allowing them to grow, take responsibility for projects and apply for funding, we increase our capacity for innovation. It also creates a ripple effect of learning and a positive atmosphere that encourages others to get involved and contribute to development. (Public urban PCC with > 10 000 registered patients)

#### Structures and processes

Managers reported promoting staff involvement by embedding innovation in everyday routines and shared forums. Some relied on existing structures, such as morning briefings, workplace meetings and planning days, to support staff in generating ideas, discussing challenges and agreeing on solutions. Others established dedicated forums for innovation and development work. While some managers described well-defined processes encompassing all stages of the innovation process, others appeared to support ideas on an ad hoc basis.

Suggestions are listened to, then presented to the management team and subsequently to all staff; we discuss pros and cons, pilot them and continuously evaluate before making a decision. (Private urban PCC with > 10 000 registered patients)

Time is made available whenever a staff member proposes a good idea. (Private non-urban PCC with < 10 000 registered patients)

Innovation was often described as improvement and development work. It was commonly understood as a collective process characterised by continuous dialogue and interprofessional collaboration, in which ideas are promptly considered, tested and iteratively refined. The need to allocate protected time – through schedule adjustments, temporary workload reduction or weekly sessions – in order to make it possible for staff to engage meaningfully in innovation work was a recurring theme.

However, some managers identified organisational and resource-related constraints as major barriers to staff involvement in innovation and development. Primary care was described as tightly bound by economic and production-oriented systems, leaving little time or space for innovation work. Because of heavy workloads, constant patient care duties and budget constraints, daily operations took priority, often leaving little time for follow-up or participation in innovation activities. Recruitment challenges and the onboarding of new staff further reduced opportunities for employee involvement.

Unfortunately, primary care is constrained by a financially driven system where production takes precedence, making it often difficult to provide sufficient time and space for these matters. (Private urban PCC with > 10 000 registered patients)

It’s not possible – we only have enough resources for day-to-day work due to budget cuts. We’re firefighting every day, and there’s ZERO time for development. (Public urban PCC with < 10 000 registered patients)

## Discussion

### Main findings

In this study, we describe innovation activities in Swedish primary care. We also explore innovation drivers, strategies and organisational culture from the perspective of primary care managers. Overall, the results indicate a strong engagement in innovation within Swedish primary care, with participating managers reporting product, process, and organisational innovations at broadly similar rates. According to managers, innovation ideas typically arose within the PCC, and the managers viewed themselves as central to the generation of these ideas. Innovation-related collaboration was predominantly internal, with limited engagement with individual patients or external partners.

Managers sought to encourage staff involvement in innovation through leadership skills and by fostering an innovation-oriented culture. They stressed the value of collaboration, both between professions and with other practices, to foster learning and development. It was noted that innovations sometimes introduced unintended effects, such as operational and administrative burdens. In such cases, it was important to reassess and, if necessary, revert the changes. Several managers described the difficulties of driving innovation in primary care, often due to organisational and resource-related limitations.

### Discussion of results and a comparison with existing literature

The findings describe the occurrence of product (goods and services), process and organisational innovations – three types previously recognised in Swedish PCCs [14]. Although the respondents in this study may represent a more innovation-oriented group than the average primary care manager, the findings indicate substantial innovation activity in Swedish primary care. This observation aligns with Sweden’s broader position as one of the Organisation for Economic Co-operation and Development (OECD) countries with the highest innovation output, particularly in terms of transforming new knowledge into practical and commercial applications [37]. It is also consistent with the Swedish government’s aim for Sweden to be a leading knowledge nation that invests in innovation and research [38]. However, the reported innovation appears to be largely ad hoc, with only half of managers reporting specific innovation objectives and even fewer systematically developing and evaluating employees’ ideas – a pattern also reflected in the qualitative analysis. Given the observed high level of innovation activity, it is noteworthy that several respondents described primary care as resource-constrained, high-pressure and focused on care production. While these conditions were largely perceived as barriers to innovation and development, some managers also viewed them as drivers of innovation aimed at improving patient care and the work environment. In fact, as many as 37% of managers reported that problems or crises requiring immediate action had prompted innovation initiatives. Moreover, the managers’ PCCs generally received limited external support for conducting innovation activities.

The findings suggest that managers view a change-oriented staff as the most critical factor enabling innovation. In addition, most innovation ideas originated from primary care managers and healthcare professionals engaged in everyday clinical practice. This finding indicates that managers regard both their own role and that of employees as central to innovation and development. This view is increasingly recognised in organisations [39] and draws attention to employee-driven innovation, in which new ideas, practices or solutions stem from employees’ first-hand experiences in their routine work [40]. Employee engagement with innovation is closely linked to managerial support [39, 41] and organisational culture [25]. The managers in this study described several strategies to promote employee involvement with innovation, including establishing trust-based leadership, delegating responsibility and enhancing meaning-making through reflection. These findings are consistent with research showing that managerial strategies that foster employee empowerment are key drivers of employee engagement in innovation [14, 42]. For example, Hansen et al. [39] underline the role of ‘empowering management’ and collaborative leadership in promoting employees’ willingness and ability to engage in innovation.

While collaboration with external actors was less commonly reported as part of the innovation process, several organisational innovations concerned formalised collaboration structures, particularly between municipalities and regions. This finding suggests that such collaboration was often implemented as an organisational arrangement rather than developed through active, ongoing co-creation with external stakeholders. Given the rapid development of digital innovations in primary care [1], it is noteworthy that collaboration with external stakeholders remains so limited. Previous research has emphasised the need for value co-creation in health innovations [43, 44] in order to ensure that new solutions deliver the key healthcare outcomes outlined in the Quadruple Aim [45], which include improved patient experience, advancing population health, controlling healthcare costs and increased clinician satisfaction [43]. Furthermore, collaboration among primary care, industry and academia is crucial for effectively evaluating the broader impacts and consequences of innovations [46] – an essential step in facilitating implementation [47] and avoiding low-value care.

Some managers pointed out that innovations did not always turn out as expected and that it was necessary to be able to step back and make adjustments in such cases. This is typical of complex organisations such as primary care, where the many interacting elements make outcomes difficult to predict, such that innovations require continuous adaptation along the way [48–50]. As Desveaux et al. [51] argue, standardised, one-size-fits-all approaches often undermine engagement, so a change-management strategy that adapts implementation to the specific context is key. Similarly, several scholars have suggested that innovations are not simply implemented but rather translated into their contexts [50, 52–55]. Considering the literature, the findings of the current study reinforce the notion that it is essential for each PCC to have sufficient autonomy to tailor innovations to its specific context, particularly when innovations are introduced through top-down processes.

### Strengths and limitations of the study

This is an exploratory study and does not claim to be exhaustive. Its mixed-methods approach is considered a strength, as it allows quantitative and qualitative findings to be considered together for a more comprehensive understanding.

The study has some limitations worth noting. It employed a cross-sectional design, so no causal claims can be made. Another limitation is the selection bias commonly associated with such designs [29]. The majority of respondents (79%) were managers from PCCs located in urban areas. It is difficult to determine how representative this is, as up-to-date national statistics on the geographic distribution of PCCs across urban, rural and remote areas are unavailable. However, 2021 statistics showed that approximately 30% of PCCs were in urban municipalities, 45% in mixed and 25% in rural municipalities [56], suggesting a potential overrepresentation of urban PCCs in the sample.

Although the overall response rate of 38% was lower than desired, it falls within the range commonly observed in comparable studies [57]. Moreover, when Statistics Sweden [32] conducted a similar survey in 2014, the response rate was 30%. Still, the modest response rate may limit the strength of the findings and may have introduced bias, with managers interested and engaged in innovation being more inclined to participate. To increase the response rate, three reminders were sent, and the questionnaire remained open online for 3 months to allow as many managers as possible to respond.

The SCB questionnaire was previously used in a primary care context [32]. The decision not to include communicative innovations in the questionnaire was informed by previous research indicating that innovation in Swedish primary care predominantly concerns service, process, and organisational innovations [14]. The exclusion was also intended to reduce respondent burden and improve the overall feasibility of the survey. While omitting one dimension of innovation may have affected the questionnaire’s content validity [58], innovation is a broad and complex phenomenon, and the present study does not claim to capture all possible dimensions of innovation within Swedish primary care.

An analysis of non-responses indicated that managers primarily did not respond to or complete the questionnaire due to limited time and other pressing responsibilities. The gradual decline in responses throughout the questionnaire supports this explanation. This reflects a widely recognised problem in healthcare research: namely, that healthcare staff often have limited time to participate in studies [59]. Moreover, the retrospective 2-year scope of the questionnaire made it difficult for recently appointed managers or those leading newly established PCCs to provide responses.

### Implications and further research

The findings of this study suggest that innovation activities within Swedish primary care are predominantly carried out through intra-organisational collaboration. This underscores the need to strengthen collabora­tion between primary care and key external stakehold­ers, including industry, academia and other areas of the healthcare sector. This perspective aligns with the rationales behind national efforts such as the Swedish SIISH project (2022–2025), which was led by seven university hospital regions and their innovation support systems [8]. The SIISH project aimed to accelerate the implementation of healthcare innovations. Several priority areas were identified, including the need to establish effective communication channels between healthcare, industry, academia and other stakeholders, and the importance of embedded and collaborative innovation strategies and processes. Such processes may include industry-facing services, such as testbeds and validation frameworks, as well as collaborative platforms where healthcare providers take a leading role [8]. Future research should explore how system-level structures for collaboration and innovation can be embedded across the Swedish healthcare system, including primary care. Such research could include examining organisational processes, governance models and digital tools that facilitate collaboration among care providers, academia and industry, and assessing the long-term sustainability and impact of such initiatives. Complexity theory, which can be used to focus on the dynamic, interconnected and adaptive nature of healthcare systems, could be a useful theoretical lens here [49, 60]. Future research could also explore how primary care managers are supported by the broader healthcare system in organising, governing and leading innovation and development.

## Conclusion

This study provided an overview of the innovation landscape in Swedish primary care by exploring innovation and its contextual conditions from the perspective of primary care managers. The findings indicate strong engagement with innovation at Swedish PCCs. Collaboration in innovation largely occurred within and between PCCs, while the involvement of external stakeholders was limited. Managers reported challenges in driving innovation due to resource constraints and high workload. Overall, while innovation activity is evident, significant barriers remain – particularly the pressures of high workload and limited external innovation-related collaboration. These findings underscore the need for system-level strategies and processes that enable cross-sector partnerships. Due to the low response rate and potential selection bias, the results should be interpreted with caution; however, they contribute important insights to the literature on innovation in primary care. 

## Supplementary Information

Below is the link to the electronic supplementary material.

Supplementary Material 1

Supplementary Material 2

Supplementary Material 3

Supplementary Material 4

Supplementary Material 5

Supplementary Material 6

## Data Availability

The datasets used and/or analysed during the current study are available from the corresponding author upon reasonable request.

## Declarations

### Description of data

This file contains the full questionnaire distributed to participants, included all items and response options.

## Abbreviations

PCC: Primary care centre
SCB: Statistics Sweden
STC: Systematic Text Condensation (STC)
