# Supplementary material for: Exploring innovation landscapes: a national cross-sectional study of Swedish primary care from the viewpoint of primary care managers
Source: BMC Health Serv Res. 2026 Jun 25;26:871. doi: 10.1186/s12913-026-14870-y (PMC13308186; doi:10.1186/s12913-026-14870-y)
Supplement: Supplementary file 1 — Supplementary Material 1 [file 12913_2026_14870_MOESM1_ESM.pdf]

Additional file 1

Background questions

1a. What is your position at the health centre?
☐ Primary care manager
☐ Deputy primary care manager
☐ Other, please specify below:

1b. How long have you held this position?

2. What is your professional background?

3. Do you hold a PhD?
☐ Yes
☐ No

4. The primary care centre is…
☐ Publicly owned
☐ Privately owned
☐ Other form of ownership

5. In which of the following areas is your primary care centre located?
Urban area – localities with more than 3000 inhabitants. This also includes areas within a 5-minute drive to the urban area.
Rural area – areas located within a 5- to 45-minute drive to an urban area with more than 3000 inhabitants.
Remote area – areas more than a 45-minute drive to the nearest urban area with more than 3000 inhabitants, as well as islands without a fixed mainland connection.
☐ Urban area
☐ Rural area
☐ Remote area

6. How many registered patients does the primary care centre have?

7. How many employees does the primary care centre have?

8. Is any member of staff at the primary care centre a PhD holder?
☐ Yes, employed at the primary care centre for the entire period 2022–2023.
☐ Yes, employed at the primary care centre for part of the period 2022–2023.
☐ No

Types of innovations

This section concerns innovations introduced by the primary care centre during 2022–2023. The questions are divided into product innovation, process innovation and organisational innovation. Each type of innovation is explained at the start of its respective section.

Product innovation

A product innovation is a new or improved good or service that differs significantly from the primary care centre's previous goods or services. To be considered an innovation, the good or service must have been made available to users. Purchases of new or significantly improved goods and services that have been introduced are also included.

Note that the good or service does not need to be new to other primary care centres, hospitals, companies or organisations.

Include digital goods and services, but exclude changes that are purely aesthetic and not expected to result in improved operations or better outcomes.

9. Did the primary care centre introduce any new or significantly improved products (goods or services) during 2022–2023 in the following areas?
Please select one option for each row.

	Yes	No	Do not know
Medical treatments	☐	☐	☐
Medical equipment and instruments	☐	☐	☐
Non-medical equipment and instruments	☐	☐	☐
Services used by patients or other citizens	☐	☐	☐
Other services or goods	☐	☐	☐
During 2022–2023, has the primary care centre conducted activities aimed at developing product innovations that have not yet been implemented?	☐	☐	☐

10. Did the primary care centre introduce new or significantly improved products (goods or services) during 2022–2023 that were…
Please select one option for each row.

	Yes	No	Do not know
New only to the primary care centre	☐	☐	☐
New both to the primary care centre and to primary care at large	☐	☐	☐

Process innovation

Process innovation refers to a new or significantly improved process for one or more functions at the primary care centre. It should differ substantially from the centre's previous processes and be implemented within the organisation.

Note that the process does not need to be new to other primary care centres, hospitals, companies or organisations.

Disregard changes, expansions of existing activities and reorganisations that are not expected to result in improved operations or better outcomes.

11. Did the primary care centre introduce any new or significantly improved processes in the following areas during 2022–2023?
Please select one option for each row.

	Yes	No	Do not know
Treatment programs or therapeutic strategies	☐	☐	☐
Diagnostic methods	☐	☐	☐
Guidelines for coordinating care around the individual patient	☐	☐	☐
Methods for involving patients and relatives in decision-making	☐	☐	☐
Methods for engaging patients in their own care	☐	☐	☐
Methods for reducing waiting times	☐	☐	☐
Support services (maintenance, procurement, logistics, etc.)	☐	☐	☐
Other methods for the production of goods and services	☐	☐	☐
During 2022–2023, has the primary care centre conducted activities aimed at developing process innovations that have not yet been implemented?	☐	☐	☐

Organisational innovation

Organisational innovation refers to a new or substantially improved method for organising operations within the primary care centre. It must differ significantly from the centre's previous methods and be implemented within the organisation itself.

Note that the method does not need to be new to other primary care centres, hospitals, companies or organisations. The organisational innovation must result from strategic decisions made by the centre's management.

Exclude changes, expansions of existing activities or restructurings that are not expected to lead to improved operations or better outcomes.

12. Did the primary care centre introduce any new or significantly improved methods for organising its operations in the following areas during the years 2022–2023?
Please select one option on each row.

	Yes	No	Do not know
Organisation of work responsibilities or decision-making	☐	☐	☐
Organisational management systems to improve efficiency and performance (e.g. Lean)	☐	☐	☐
Systems for collecting and processing knowledge and information	☐	☐	☐

(e.g. quality registers)			
Training or education systems for staff or management	☐	☐	☐
Measures to reduce the administrative burden on healthcare staff	☐	☐	☐
Collaboration between municipalities and regions	☐	☐	☐
Other methods for organising work (e.g. new ways of working)	☐	☐	☐
During 2022–2023, did you carry out activities aimed at developing organisational innovations that have not yet been implemented?	☐	☐	☐

Effects of innovation

The questions in this section concern all innovations (product, process and organisational) that were introduced at the primary care centre during 2022–2023.

13. What were the effects of the innovations in the following areas?

	Positive effect	No effect	Negative effect	Do not know
Ability to provide care to a broader patient group	☐	☐	☐	☐
Faster and/or better recovery for patients	☐	☐	☐	☐
More effective treatment of patients	☐	☐	☐	☐
Shorter treatment time for patients	☐	☐	☐	☐
Reduced suffering for patients	☐	☐	☐	☐
Improved patient safety	☐	☐	☐	☐
Patients’ access to information	☐	☐	☐	☐
Increased autonomy for patients/relatives	☐	☐	☐	☐
Increased satisfaction among staff	☐	☐	☐	☐
Reduced administrative burden for healthcare staff	☐	☐	☐	☐
Efficient use of resources	☐	☐	☐	☐
Cost reduction	☐	☐	☐	☐
Increased access to information (about patients, clinical studies)	☐	☐	☐	☐
Other effect, please specify below:	☐	☐	☐	☐

Support for innovation

The questions in this section concern all innovations (product, process and organisational) that were introduced at the primary care centre during 2022–2023.

14. Approximately how many innovations has the primary care centre introduced during 2022–2023?

15. Did the primary care centre receive external support for innovation activities from any of the following actors during 2022–2023?
Include only support specifically earmarked for innovations.

	Yes	No	Do not know
Municipality or region	☐	☐	☐
VINNOVA, ALMI, research councils	☐	☐	☐
Other government agencies	☐	☐	☐
EU or other international sources	☐	☐	☐
Private foundations or interest organisations	☐	☐	☐
Other, please specify below:	☐	☐	☐

Drivers and strategies for innovation

The questions in this section concern all innovations (product, process and organisational) that were introduced at the primary care centre during 2022–2023.

16. Which of the following reasons contributed to innovations being initiated at the primary care centre during 2022–2023?

	Yes	No	Do not know
a) Requests from patients/relatives or patient organisations	☐	☐	☐
b) Requests from healthcare staff	☐	☐	☐
c) Implementation of new laws or regulations	☐	☐	☐
d) Implementation of new regional or municipal policies or priorities	☐	☐	☐
e) A problem or crisis that required immediate action	☐	☐	☐
f) Requirements to implement new IT systems	☐	☐	☐
g) Innovations at other primary care centres	☐	☐	☐
h) Internal reorganisation	☐	☐	☐
i) Reduction in the primary care centre's budget	☐	☐	☐
j) Increase in the primary care centre's budget	☐	☐	☐
k) Other, please specify below:	☐	☐	☐

17. Which of the above reasons was the most important? *Please indicate the letter.*

- ☐ a
- ☐ b
- ☐ c
- ☐ d
- ☐ e
- ☐ f
- ☐ g
- ☐ h
- ☐ i
- ☐ j
- ☐ k

18. Which of the following actors contributed the ideas for the innovations at the primary care centre?

	Yes	No	Do not know
a) Management of the primary care organisation	☐	☐	☐
b) Management of the primary care centre	☐	☐	☐
c) Physicians	☐	☐	☐
d) Nurses	☐	☐	☐
e) Assistant nurses	☐	☐	☐
f) Rehabilitation staff	☐	☐	☐
g) Other healthcare staff	☐	☐	☐
h) Administrative staff	☐	☐	☐
i) Support staff (e.g. cleaning, maintenance)	☐	☐	☐
j) Patients or relatives	☐	☐	☐
k) Other primary care centres	☐	☐	☐
l) Private companies	☐	☐	☐
m) Universities or public research organisations	☐	☐	☐
n) Regional politicians	☐	☐	☐
o) Other, please specify below:	☐	☐	☐

19. Which of the above actors was the most important? *Please indicate the letter.*

- ☐ a
- ☐ b
- ☐ c

☐ d

☐ e

☐ f

☐ g

☐ h

☐ i

☐ j

☐ k

☐ l

☐ m

☐ n

☐ o

20. Did your primary care centre have any collaboration on innovations with any of the following actors during 2022–2023?
Collaboration on innovations refers to active cooperation with companies, public organisations or other actors. Exclude work that is outsourced under contract where no active collaboration takes place.

	Yes	No	Do not know
a) Units within your primary care centre	☐	☐	☐
b) Staff groups at your primary care centre	☐	☐	☐
c) Other primary care centres and healthcare organisations	☐	☐	☐
d) Patient organisations	☐	☐	☐
e) Other non-profit or non-governmental organisations	☐	☐	☐
f) Universities or public research organisations	☐	☐	☐
g) Private sector, consultants or business organisations	☐	☐	☐
h) Individual patients	☐	☐	☐
i) Other, please specify below:	☐	☐	☐

21. Which of the above actors was the most important? *Please indicate the letter.*

☐ a

☐ b

☐ c

☐ d

☐ e

☐ f

☐ g

☐ h

☐ i

Innovation culture and organisation

22. During 2022–2023, did your primary care centre have any of the following?

	Yes	No	Do not know
Specific goals for innovation activities	☐	☐	☐
An innovation department or unit	☐	☐	☐
A system for evaluating and developing innovative ideas submitted by employees	☐	☐	☐
A system for evaluating and introducing new medicines or treatments	☐	☐	☐
Sufficient resources (time, money, expertise) to develop an innovation	☐	☐	☐
Assigned specific individuals to take an innovation from idea to completion	☐	☐	☐

23. Does your primary care centre involve…

	Yes	No	Do not know
Patients/relatives in identifying problems and potential solutions	☐	☐	☐
Patients/relatives in testing and introducing innovations	☐	☐	☐

24. To what extent did the following conditions contribute to facilitating innovation work during 2022–2023?

	To a great extent	To some extent	Not at all	Do not know
Change-oriented primary care management at the <u>political level</u>	☐	☐	☐	☐
Change-oriented primary care management at the <u>administrative/ official level</u>	☐	☐	☐	☐
Change-oriented staff	☐	☐	☐	☐
Effective collaboration with other	☐	☐	☐	☐

primary care centres				
Effective collaboration with hospital care	☐	☐	☐	☐
Effective collaboration with municipal care	☐	☐	☐	☐
Effective coordination with local and regional authorities	☐	☐	☐	☐
Wide access to expert knowledge	☐	☐	☐	☐
Other, please specify below:	☐	☐	☐	☐

Primary care centre's most significant innovation

The following questions concern the primary care centre's most significant innovation – the innovation that has created the greatest benefit during 2022–2023. The innovation must have been introduced during this period, but the activities that enabled it may have been carried out earlier.

25. Please briefly describe the primary care centre's most significant innovation introduced during 2022–2023.

26. Which actor(s) contributed the idea for this innovation?
☐ Management of the primary care organisation
☐ Management of the primary care centre
☐ Physicians
☐ Nurses
☐ Assistant nurses
☐ Rehabilitation staff
☐ Other healthcare staff
☐ Administrative staff
☐ Support staff (e.g. cleaning, maintenance)
☐ Patients or relatives
☐ Other primary care centres
☐ Private companies
☐ Regional politicians
☐ Universities or public research organisations
☐ Other, please specify below:

27. Which actor(s) contributed the most to the development of this innovation?
☐ Management of the primary care organisation
☐ Management of the primary care centre
☐ Physicians
☐ Nurses
☐ Assistant nurses
☐ Rehabilitation staff
☐ Other healthcare staff
☐ Administrative staff

☐ Support staff (e.g. cleaning, maintenance)
☐ Patients or relatives
☐ Other primary care centres
☐ Private companies
☐ Regional politicians
☐ Universities or public research organisations
☐ Other, please specify below:

28. Who is/are affected by this innovation?
☐ Management of the primary care centre
☐ Physicians
☐ Nurses
☐ Assistant nurses
☐ Rehabilitation staff
☐ Other healthcare staff
☐ Administrative staff
☐ Support staff (e.g. cleaning, maintenance)
☐ Patients
☐ Relatives
☐ Other, please specify below:

29. What was the purpose of this innovation? *(multiple answers possible)*
☐ To replace a previous service, product, process or organisational method
☐ To offer an entirely new service, product, process or organisational method
☐ Cost savings
☐ Quality improvements
☐ To reduce the administrative burden on healthcare staff
☐ To create greater value for patients
☐ Other, please specify below:

30. As primary care centre manager, how do you create conditions for your staff to be involved in innovation and development at the workplace?
