# Supplementary material for: Exploring innovation landscapes: a national cross-sectional study of Swedish primary care from the viewpoint of primary care managers
Source: BMC Health Serv Res. 2026 Jun 25;26:871. doi: 10.1186/s12913-026-14870-y (PMC13308186; doi:10.1186/s12913-026-14870-y)
Supplement: Supplementary file 2 — Supplementary Material 2 [file 12913_2026_14870_MOESM2_ESM.pdf]

Additional file 2

Reasons for non- or partial response reported by managers (multiple answers possible).

Reasons for non- or partial response	Number of responses *n* (%)	Examples of respondents' comments
Time constraints and priorities	51 (58.6)	The reason is a very pressured work situation where, at the moment, I'm juggling everything at once.<br><br>In the last 10 days, I've received five different questionnaires that I'm expected to complete. When am I supposed to find time to work?<br><br>Unfortunately, I prioritise tasks that I am obliged to do or that are directly relevant to my work.<br><br>As you probably know, healthcare is under significant pressure and overtime is extensive, so your email hasn't been prioritised because daily operations come first.
New manager or newly established PCC	12 (14.5)	I'm completely new in my role and don't really have the time or knowledge to participate at the moment.<br><br>As I have only been acting manager for a few weeks, it is difficult for me to answer these questions … and I do not have the time right now to look back at 2022–2023 …
Comprehension issues	8 (9.2)	I started the survey but withdrew because I didn't understand the questions.
Regional focus	6 (6.9)	I didn't answer simply because I'm not employed in the Västra Götaland region.<br><br>Why did I get this email in the Stockholm region?
Declined without reason	6 (6.9)	
No innovation activity/nothing to contribute	3 (3.4)	…during the period 2022–2023, no innovations were introduced…
Previous lack of feedback from other surveys	1 (1.1)	…and we never see any results from our participation either.
