# Supplementary material for: Exploring innovation landscapes: a national cross-sectional study of Swedish primary care from the viewpoint of primary care managers
Source: BMC Health Serv Res. 2026 Jun 25;26:871. doi: 10.1186/s12913-026-14870-y (PMC13308186; doi:10.1186/s12913-026-14870-y)
Supplement: Supplementary file 3 — Supplementary Material 3 [file 12913_2026_14870_MOESM3_ESM.pdf]

Additional file 3

Example illustrating the different steps of the qualitative analysis using STC [35].

STC step	Example from the data
1. Total impression	Examples of preliminary themes included leadership, collaboration, barriers to innovation and innovation in daily practice.
2. Identifying meaning units, codes and subcodes	'I am highly supportive of change initiatives and strive to maintain an open and responsive attitude towards suggestions and ideas from staff.'<br><br>Code:<br>Leadership and culture<br><br>Subcodes:<br>Supportive and change-oriented leadership<br>Responsiveness to employee suggestions
3. Condensation	Supportive of change and receptive to staff suggestions
4. Synthesising	Several managers stated that they valued innovation and development, and many sought to encourage employee involvement by inviting ideas and showing interest in employees' suggestions.
