# Supplementary material for: Exploring innovation landscapes: a national cross-sectional study of Swedish primary care from the viewpoint of primary care managers
Source: BMC Health Serv Res. 2026 Jun 25;26:871. doi: 10.1186/s12913-026-14870-y (PMC13308186; doi:10.1186/s12913-026-14870-y)
Supplement: Supplementary file 4 — Supplementary Material 4 [file 12913_2026_14870_MOESM4_ESM.pdf]

## Additional file 4

Support for innovation.

| External support for innovation | *n* (%) | Yes *n* (%) | No *n* (%) | Do not know *n* (%) |
|---|---|---|---|---|
| *Did the primary care centre receive external support for innovation activities from any of the following actors during 2022–2023?* | | | | |
| Municipal or regional authority | 298 | 99 (33.2) | 189 (63.4) | 10 (3.4) |
| VINNOVA, ALMI, research council | 298 | 9 (3.0) | 266 (89.3) | 23 (7.7) |
| Other government agencies | 298 | 29 (9.7) | 252 (84.6) | 17 (5.7) |
| The EU or other international sources | 298 | 5 (1.7) | 273 (91.6) | 20 (6.7) |
| Private foundations or interest organisations | 298 | 6 (2.0) | 274 (91.9) | 18 (6.0) |
| Other, please specify below | 298 | 14 (4.7) | 240 (80.5) | 44 (14.8) |
