# Supplementary material for: Exploring innovation landscapes: a national cross-sectional study of Swedish primary care from the viewpoint of primary care managers
Source: BMC Health Serv Res. 2026 Jun 25;26:871. doi: 10.1186/s12913-026-14870-y (PMC13308186; doi:10.1186/s12913-026-14870-y)
Supplement: Supplementary file 5 — Supplementary Material 5 [file 12913_2026_14870_MOESM5_ESM.pdf]

Additional file 5

Innovation culture, organisation and patient involvement in innovation.

Innovation culture and organisation	*n*	Yes<br>*n* (%)	No<br>*n* (%)	Do not know<br>*n* (%)
In 2022–2023, did your PCC have…				
Specific objectives for innovation activities	261	133 (51.0)	114 (43.7)	14 (5.4)
An innovation department or innovation unit	261	22 (8.4)	223 (85.4)	16 (6.1)
A system for evaluating and developing innovative ideas proposed by employees	261	98 (37.5)	143 (54.8)	20 (7.7)
A system for evaluating and introducing new medicines or treatments	261	61 (23.4)	180 (69.0)	20 (7.7)
Sufficient resources (time, funding, expertise) to develop an innovation	261	76 (29.1)	159 (60.9)	26 (10.0)
Assigned specific individuals to take an innovation from the idea stage to completion	261	135 (51.7)	110 (42.1)	16 (6.1)
Does your PCC involve…				
Patients/relatives to identify problems and possible solutions	261	83 (31.8)	157 (60.2)	21 (8.0)
Patients/relatives involved in testing and introduction of innovations	261	63 (24.1)	178 (68.2)	20 (7.7)
