# Supplementary material for: Exploring innovation landscapes: a national cross-sectional study of Swedish primary care from the viewpoint of primary care managers
Source: BMC Health Serv Res. 2026 Jun 25;26:871. doi: 10.1186/s12913-026-14870-y (PMC13308186; doi:10.1186/s12913-026-14870-y)
Supplement: Supplementary file 6 — Supplementary Material 6 [file 12913_2026_14870_MOESM6_ESM.pdf]

Additional file 6

Conditions for innovation work.

Innovation culture and organisation	*n*	High degree *n* (%)	Some degree *n* (%)	Not at all *n* (%)	Do not know *n* (%)
To what extent did the following conditions facilitate innovation work during the years 2022–2023?					
Change-oriented primary care management at political level	261	33 (12.6)	76 (29.1)	108 (41.4)	44 (16.9)
Change-oriented primary care management at administrative level	261	62 (23.8)	81 (31.0)	77 (29.5)	41 (15.7)
Change-oriented staff	261	173 (66.3)	70 (26.8)	5 (1.9)	13 (5.0)
Effective collaboration with other PCCs	261	54 (20.7)	117 (44.8)	66 (25.3)	24 (9.2)
Effective collaboration with hospital care	261	20 (7.7)	67 (25.7)	148 (56.7)	26 (10.0)
Effective collaboration with municipal care	261	45 (17.2)	100 (38.3)	92 (35.2)	24 (9.2)
Effective coordination with local and regional authorities	261	19 (7.3)	55 (21.1)	150 (57.5)	37 (14.2)
Extensive access to expert knowledge	261	43 (16.5)	85 (32.6)	99 (37.9)	34 (13.0)
Other	261	9 (3.4)	9 (3.4)	86 (33.0)	157 (60.2)
